# Pattern-IT: A method for mapping stakeholder engagement with complex systems

**DOI:** 10.1016/j.mex.2020.101123

**Published:** 2020-11-02

**Authors:** Hannah Devine-Wright

**Affiliations:** a Trinity College Dublin, Dublin College Green, Dublin 2 DO2 PN40, Ireland; b Placewise Ltd, Uffculme, Devon EX15 3DR, UK

**Keywords:** Card sorting, Mapping sentences, Multiple sorting task, Co-creation, Workshop, Stakeholder engagement

## Abstract

Pattern-IT is a participatory, card sorting activity that aims to illuminate the relationships between people, technologies and concepts in complex systems. Pattern-IT combines two methods: card sorting [11] and mapping sentences [2,6]. Depending on the aims and scope of the research or topic, Pattern-IT can be used in an exploratory, descriptive, or interpretative manner. It is a co-created, adaptable and enjoyable method that can be used with individuals or groups, in-person or online, with or without facilitation. In this paper, Pattern-IT was conducted face-to-face in a moderated group setting using physical cards to explore engagement by project partners with publics involved in implementing Smart Local Energy Systems (SLES). SLES are decentralised energy systems that use information communication technologies (e.g. smart meters, blockchain, real-time pricing) to connect low carbon energy generation (e.g. solar PV) with energy storage and energy services (e.g. electric vehicles).

• A method that combines card sorting and mapping sentences.
• Uses mapping sentences as an organising and data generating tool.
• A novel co-created participatory method for use with individuals or groups.

## Graphical abstract

**Figure:**
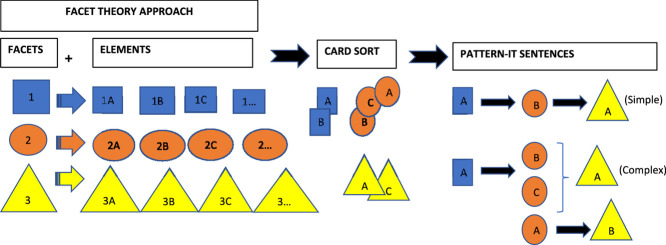

Specifications Table

**Table:** 

Subject Area:	Psychology
More specific subject area:	Environmental Psychology
Method name:	Pattern-IT
Name and reference of original method:	Pattern-IT is a participatory, card sorting procedure that combines two methods: card sorting [11] and mapping sentences [2]. Stephenson [11]. Correlating persons instead of tests. *J. Pers.* 4 (1) (1935), 17–24. Canter [2] The potential of Facet Theory for applied social psychology. *Qual. Quant.* 17, (1983), 35–67.

## Method details

Pattern-IT is a participatory, card sorting procedure that combines two methods: card sorting [3], [11] and mapping sentences [2,6]. Pattern-IT is an adaptable method that can be used to illuminate the relationships between people, technologies and concepts in complex systems (for example, energy, health, education, food or justice systems). This paper includes examples from Pattern-IT conducted with academics, companies, local authorities and community organisations seeking to understand and inform public engagement strategies associated with Smart Local Energy Systems (SLES). SLES are decentralised energy systems that use information communication technologies (e.g. smart meters, blockchain, real-time pricing) to connect low carbon energy generation (e.g. solar PV) with energy storage and energy services (e.g. electric vehicles).

Pattern-IT is a co-created, adaptable and enjoyable card sorting method that can be used with individuals or groups, in-person or online, with or without facilitation [1], [4], [8]. In this paper, Pattern-IT is conducted face-to-face using physical cards inscribed with text. Unlike existing card sorting methods (e.g. Q-methodology, multiple sorting tasks) that generate interval or ordinal data, Pattern-IT generates ‘sentences’ that can be ‘read’ in situ by participants, or analysed further using methods such as content or discourse analysis.

The Pattern-IT method is detailed below as a series of 13 steps that correspond to three research phases: preparation, implementation and interpretation. Each step is conducted by a researcher with workshop facilitation and qualitative data analysis skills.

## Preparation phase

 

### Specify the facets

The researcherx specifies a number of facets depending on the topic of study [2], [9], [10]. Pattern-IT applied to SLES used five of six generic facets*: Actors* (people and/or organisations to engage with), *Actions* (ways of engaging), *Impacts* (change over time), *Outcomes* (actual and/or anticipated) and *Methods* (tools and techniques). A sixth generic facet is *Evaluation* (measures).

### Specify the process by creating a mapping sentence (optional)

A priori, the researcher develops an idea of how the facets relate to each other [2], [5], [7]. This can be achieved by creating a mapping sentence (or Theory of Change) which specifies an order for the facets. In the SLES example, it was hypothesised that *Actions* (ways of engaging) would precede *Actors* which would lead to *Impacts* (change) resulting in *Outcomes* when using specific *Methods*.

### Differentiate each facet

The researcher allocates a colour to each facet, similar to a suit within a deck of cards. To aid differentiation it is preferable to select higher contrast colours (orange, blue, yellow, grey, white and black) and avoid using green. Differentiation can be aided by printing the name of the facet in the corner of each card. For example, *Actors* = pink, *Actions* = dark yellow, *Impacts* = red, *Outcomes* = pale yellow, *Methods* = green, and *Evaluation* = purple.

### Identify elements within each facet

Each facet is made up of elements which can be pre-defined by the researcher and/or generated by participants. In this example, the researcher specified 17 *Actions* (e.g. educating, incentivising, modelling), 7 *Actors* (e.g. customers, intermediaries, local council), 3 *Impacts* (increase, decrease, does not change), 17 *Outcomes* (e.g. air quality, carbon emissions, fuel poverty) and 26 *Methods* (e.g. advertising, smartphone apps, surveys) that were relevant for understanding engagement with SLES.

### Create a card for each element

The researcher ascribes each of the elements to a card of the appropriate colour e.g. a pink card for ‘customer’ as it is an element with the *Actor* facet. Blank cards of each colour are included so that participants can create their own elements during Pattern-IT.

## Implementation phase

 

### Introduction and informed consent

Participants do not require any prior experience or training to use Pattern-IT. The researcher provides an introduction for participants that explains the purpose of the activity, provides a description of the Pattern-IT method, and outlines any ethical guidelines or data regulations relevant to the study, for example, informed consent and General Data Protection Guidelines (GDPR).

### Randomise the presentation of elements within each facet

The researcher randomizes the presentation of the elements (e.g. by shuffling the cards) and presents each facet as a pile of cards or, space permitting, an array of cards of the same colour laid out in close proximity on a table.

### Provide an example of a simple mapping sentence

If the order of the facets is pre-defined, a simple mapping sentence is created by the researcher who selects, or ask the participants to select, one card of each colour, that is, one element from each facet. These are placed in the specified order specified and ‘read’ like a sentence. For example, Engaging (*Action – Dark Yellow*) with Householders (*Actor - Pink*) leads to a Decrease (*Impact - Red*) in Fuel Poverty (*Outcome – Pale Yellow*) using Exhibitions (*Method - Green*).

If the order the order of the facets is not pre-defined, Pattern-IT is exploratory. The researcher explains that a simple mapping sentence can be created by selecting one element from each facet and placing them in a line so that they can be read from left to right like a sentence. The researcher asks participants to select one card of each colour and place them in a line so that they can be ‘read’ like a sentence. For example, Exhibitions (*Method - Green*) Decrease (*Impact - Red*) Fuel Poverty (*Outcome – Pale Yellow*) when Engaging (*Action – Dark Yellow*) with Householders (*Actor - Pink*).

### Create a simple mapping sentence

Participants are asked to select one element from each facet that ‘go together’, that is, makes sense to them. If required, participants can create a new element by writing the name of the missing element on a blank card of the appropriate colour. The researcher explains that there are no right or wrong sentences and no minimum or maximum number of sentences.

In the SLES study, an *Actor* was matched to an *Action* using the prompt, ‘How do you approach (or engage with) with this actor’? Participants then selected or created an *Action, Impact, Outcome* and *Method* they associated with this *Actor*.

### Pattern-IT: create simple or complex mapping sentences

A simple mapping sentence is created when one element is selected from each facet. However, in practise, participants create complex mapping sentences in several ways:

• Including multiple elements from one or more facets.

**Fig. 1:**
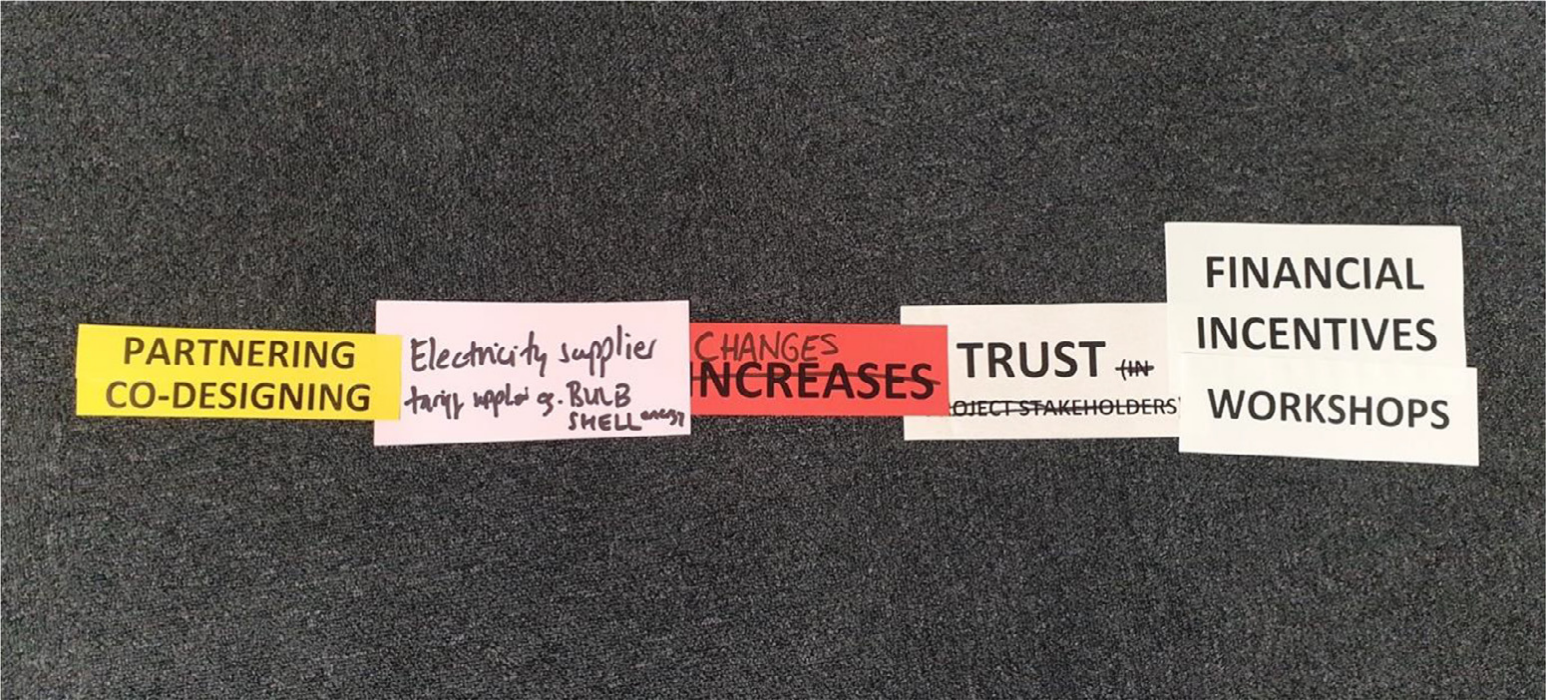
An example of a simple Pattern-IT mapping sentence (from left to right): Actions, Actors, Impact, Outcomes and Methods.

Additional elements can be pre-specified by the researcher or created (or modified) by the participants. When multiple elements are selected the elements are placed above each other in a column (see Fig. 1).

For example, ‘Engaging’ and ‘Partnering’ (*Actions – Dark Yellow*) with ‘Householders’ (*Actor - Pink*) leads to a ‘Decrease’ (*Impact - Red*) in ‘Fuel Poverty’ and ‘Carbon Emissions’ (*Outcomes – Pale Yellow*) using ‘Exhibitions’ and ‘Financial Incentives’ (*Methods - Green*).

• Including multiple facets.

For example, ‘Engaging’ (*Action – Dark Yellow*) with ‘Householders’ (*Actor - Pink*) leads to a ‘Decrease’ (*Impact - Red*) in ‘Fuel Poverty’ (*Outcome – Pale Yellow*) and an ‘Increase’ (*Impact – Red*) in ‘Energy Equity’ *(Outcome – Pale Yellow)* using ‘Exhibitions’ (*Method - Green*).

• Including a combination of multiple elements and multiple facets.

For example, ‘Engaging’ and ‘Partnering’ (*Actions – Dark Yellow*) with ‘Householders’ (*Actor - Pink*) leads to a ‘Decrease’ (*Impact - Red*) in ‘Fuel Poverty’ and ‘Carbon Emissions’ (*Outcomes – Pale Yellow*) and an ‘Increase’ (*Impact – Red*) in ‘Energy Equity’ *(Outcome – Pale Yellow)* using ‘Exhibitions’ and ‘Financial Incentives’ (*Methods - Green*).

• Modifying the order of facets if they have been pre-defined.

For example, ‘Home Visits’ *(Method – Orange)* with ‘Householders’ (*Actor - Blue*) ‘Increases’ (*Impact – Red*) ‘Engagement’ and ‘Partnering’ (*Actions - Yellow*) and ‘Decreases’ (*Impact - Red*) ‘Fuel Poverty’ and ‘Carbon Emissions’ (*Outcomes - Green*) using ‘Exhibitions’ and ‘Financial Incentives’ (*Methods - Green*).

Fig. 1 is an example of a relatively simple Pattern-IT mapping sentence created by SLES stakeholders. In Fig. 1 there are two pre-printed *Actions (Dark Yellow cards)*: ‘Partnering’ and ‘Co-designing’, these form a column on the left-hand side; one participant generated *Actor (Pink card):* ‘Electricity Supplier’; a participant modified *Impact (Red card)*: ‘Increases’ crossed out and replaced by ‘Changes’; a single *Outcome (Pale Yellow)*: ‘Trust’, and two pre-printed *Methods (Green):* ‘Financial Incentives’ and ‘Workshops’. In Fig. 1, multiple *Action (Dark Yellow)* elements are connected to a single *Actor (Pink)* element.

**Fig. 2:**
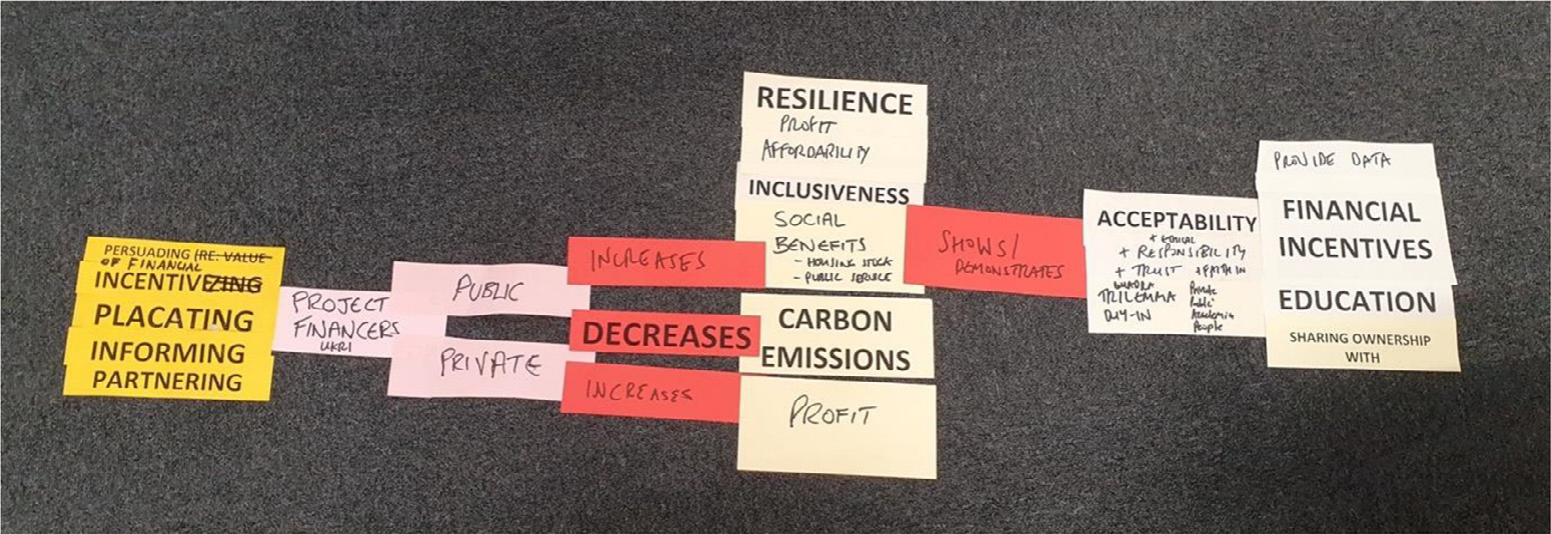
An example of a complex Pattern-IT mapping sentence (from left to right): Actions, Actors, Impacts, Outcomes, Impact, Outcomes, Methods.

Fig. 2 is an example of a complex Pattern-IT mapping sentence created by SLES stakeholders. In this example, there is a column on the left-hand side consisting of multiple pre-printed *Actions (Dark Yellow)*, a participant generated *Actor (Pink*): ‘Project Financiers’ specified further as ‘Public’ and ‘Private’. Each of the two *Actors*: ‘Public Project Financiers’ and ‘Private Project Financiers’ forms a separate row within the mapping sentence.’ Public Project Financiers’ were linked two *Impacts (Red)*: ‘Increases’ and ‘Decreases’ whilst ‘Private Project Financiers’ were linked to a single *Impact (Red*): ‘Increases’. ‘Public Project Financiers’ increased five *Outcomes (Pale Yellow)* and decreased one *Outcome (Pale Yellow)*: ‘Carbon Emissions’. ‘Private Project Financiers’ were linked to one *Impact (Red)*: ‘Decreases’ and one *Outcome (Pale Yellow):* ‘Profit’.

Fig. 2 shows that participants created a new *Impact (Red):* ‘Shows/demonstrates’. This was placed in the same row and therefore was associated with ‘Public Project Financiers’. ‘Shows/demonstrates’ was placed in a new position, after rather than before a specific, participant created *Outcome (Pale Yellow):* ‘Social Benefits’*.* Furthermore, ‘Shows/demonstrates’ was linked to multiple pre-printed and participant generated *Outcomes (Pale Yellow)* including ‘Acceptability’ and ‘Trust’. The Pattern-IT mapping sentence was completed with a column consisting of four *Methods (Green*) which included one participant generated *Method (Green):* ‘Provide Data’.

### Repeat previous

The Pattern-IT procedure is repeated as often as required or permitted. A variety of factors can influence the quantity of mapping sentences created. These include: the number of people in the group, the amount of time allocated to the activity and the clarity of instructions provided to participants. The quantity of Pattern-IT mapping sentences that are created will vary. Across four SLES workshops, participants created 5, 6, 10 and 12 mapping sentences respectively.

### Data recording

Participants are invited by the researcher to confirm that the cards are in the most suitable arrangement, one that makes sense to them, and if necessary, fix the cards into their final position, for example, using adhesive. If Pattern-IT is conducted using physical cards, create a digital record using a photographic device. Transcribe the mapping sentences by ascribing a different typeface to each facet. For example, using **Actions** (**bold**), *Actors* (*italics*), IMPACTS (UPPER CASE), Outcomes (regular text) and Methods (underlined), the mapping sentence in Fig. 1 can be written as:

**Partnering/co-designing** *Electricity suppliers* CHANGES Trust [using] Financial incentives/workshops

## Interpretation phase

 

### Data analysis

As Pattern-IT is a co-created method, the importance attached to the outcomes, i.e. the quantity or complexity of sentences, will vary. In some applications, taking part in Pattern-IT and co-creating sentences will be sufficient in itself (e.g. as a group bonding exercise). At other times, sentences will be read and recorded as they are (e.g. as a means of describing person-system relationships). Alternatively, supplementary quantitative or qualitative analysis will be conducted (e.g. to identify gaps in understanding). Suitable analytic procedures for use with Pattern-IT data include quantitative or qualitative content analysis and thematic analysis using appropriate software such as NVivo.

### Repeat Pattern-IT (optional)

In accordance with the aims of the research, Pattern-IT can be re-run with the same or different participants at another point in time. For example, participants in the SLES workshops requested that Pattern-IT was repeated annually in order to illustrate how stakeholders and stakeholder engagement practices evolve over time.

## Declaration of Competing Interest

The author declares that they have no known competing financial interests or personal relationships that could have appeared to influence the work reported in this paper.

## Appendix Supplementary materials

Image, application 1
