# Supplementary material for: Pattern-IT: A method for mapping stakeholder engagement with complex systems
Source: MethodsX. 2020 Nov 2;7:101123. doi: 10.1016/j.mex.2020.101123 (PMC7683329; doi:10.1016/j.mex.2020.101123)
Supplement: Supplementary file 1 [file mmc1.docx]

**Supplementary material *and/or* Additional information:**

Pattern-IT combines aspects of two methods: card sorting (Stephenson, 1935) and mapping sentences derived from Facet Theory (Canter, 1983).

Card sorting, in the form of Q-sorts, or Q-methodology as it became known, originated eighty-five years ago (Stephenson, 1935; 1952). Q-sorts generate normally distributed data that is analysed using parametric statistical procedures such as Factor Analysis. Cataldo et al., (1970) adapted the card sort as a survey interview method and in doing so generated categorical data suitable for non-parametric statistical analysis. Pattern-IT uses the card sorting procedure to generate sentences that can be ‘read’ as they are, or analysed using qualitative analysis such as content or thematic analysis.

Pattern-IT addresses an acknowledged failure to link the card sorting method to a theoretical framework (Conrad & Tucker, 2019) through its use of mapping sentences derived from Facet Theory (Canter, 1983). Mapping sentences are short, flexible, structured statements consisting of facets - distinct, superordinate categories which describe key components or stages in a process (Levy, 1976; Maslovaty et al., 2001), and elements - words or images, that together describe what is, or is not, known about a topic. Within psychology, mapping sentences are typically used to construct the formal definitional framework that guides the design of research or the testing of a theory (Guttman & Greenbaum, 1998). In the application of Pattern-IT described in this paper, a mapping sentence is used (in optional Step 2) to pre-define facets in much the same way as categories are pre-defined in a closed card sort. However, unlike a typical closed card sort, Pattern-IT focuses on the links and relationships, that is the patterns, created by the juxtaposition of categories rather than the similarity of content within categories. Participants have the flexibility to make simple or complex Pattern-IT sentences. Therefore, Pattern-IT can be exploratory, descriptive, or interpretative depending on the purpose of the research.

Mapping sentences are typically constructed using between four and six facets. One facet usually designates the population of respondents, that is, the actors or people involved (Levy, 1976). The remaining facets vary depending on the topic or focus of the research. The role, importance or relationship between the facets can be theorised through a priori construction of a mapping sentence by the researcher, or co-constructed in practice (Hackett, 2019). In this paper, the focus is on mapping sentences as co-constructed outcomes. This is especially important when research focuses on engagement with abstract, remote, or rarely encountered concepts or technologies.

Each facet within a mapping sentence contains sub-categories called elements (Canter, 1983). These are discrete items that can be specified a priori by the researcher and/or in situ by the participant(s). With Pattern-IT each element is ascribed to a single card with elements within the same facet ascribed to cards of the same colour. When Pattern-IT is used with physical cards containing elements described with words or images, it provides a way to explore how experience is created and organised through engagement with tangible research artefacts (Coleman, 2015). The number of elements within each facet can vary. In theory, there is no limit to how many elements each facet can contain. However, there may be practical constraints, for example, space availability. During Pattern-IT participants create their own elements or reject elements proposed by the researcher, consequently the number of elements in each facet can change over time.

Pattern-IT can be used by individuals or groups. Like card sorting more generally, Pattern-IT is an enjoyable and engaging method that enables participants to proceed at their own pace in the co-creation of rich, multi-sensory and meaningful data. As a facilitated group activity, Pattern-IT enables collective sorting decisions to be recorded in multiple formats: physically and visually as an arrangement of cards, and verbally, as real-time conversation between participants. Audio recording (with consent) of participant commentary can be used to understand how participants construct and negotiate meaning. For example, by transcribing audio recordings and conducting a thematic analysis using NVivo software. In this paper, analytic procedures are not specified as the type of analysis undertaken will depend on the goals of the project/activity under examination.

Ethical approval was attained from the University of Exeter to conduct Pattern-IT during four workshops attended by SLES project partner involved in early stage SLES projects, that is, between three- and six months post-funding award. Each of the SLES workshops was facilitated by the author, an experienced social scientist. Following a brief introduction to the workshop, all the participants provided written, informed consent for anonymised data to be collected, analysed and reported. Pattern-IT was conducted as a group activity, face-to-face, using a large table to lay out the cards. During the course of the SLES workshops, cards were turned over and re-used. In this sense, Pattern-IT is performative as it encourages the creation and organisation of experience (Coleman, 2015; Anderson, 2019). Participants enjoyed and valued Pattern-IT as a team activity that enhances social learning by enabling disparate project partners to work through complex and ambiguous issues, identify and clarify shared goals and co-construct actionable outcomes. Across four workshops, SLES project partners used the Pattern-IT method to create mapping sentences to describe and inform their stakeholder communication and engagement strategies. Participants requested that Pattern-IT was repeated annually as a longitudinal research measure to record changes in engagement with SLES stakeholders over time.
